# Overdiagnosis in breast cancer screening: the importance of length of observation period and lead time

**DOI:** 10.1186/bcr3427

**Published:** 2013-05-16

**Authors:** Stephen W Duffy, Dharmishta Parmar

**Affiliations:** 1 Policy Research Unit in Cancer Awareness, Screening and Early Diagnosis, Centre for Cancer Prevention, Wolfson Institute of Preventive Medicine, Barts and the London School of Medicine and Dentistry, Queen Mary, University of London, Charterhouse Square, London, EC1M 6BQ, UK

## Abstract

**Background:**

Overdiagnosis in breast cancer screening is a controversial topic. One difficulty in estimation of overdiagnosis is the separation of overdiagnosis from lead time that is the advance in the time of diagnosis of cancers, which confers an artificial increase in incidence when a screening programme is introduced.

**Methods:**

We postulated a female population aged 50-79 with a similar age structure and age-specific breast cancer incidence as in England and Wales before the screening programme. We then imposed a two-yearly screening programme; screening women aged 50-69, to run for twenty years, with exponentially distributed lead time with an average of 40 months in screen-detected cancers. We imposed no effect of the screening on incidence other than lead time.

**Results:**

Comparison of age- and time-specific incidence between the screened and unscreened populations showed a major effect of lead time, which could only be adjusted for by follow-up for more than two decades and including ten years after the last screen. From lead time alone, twenty-year observation at ages 50-69 would confer an observed excess incidence of 37%. The excess would only fall below 10% with 25 years or more follow-up. For the excess to be nullified, we would require 30 year follow-up including observation up to 10 years above the upper age limit for screening.

**Conclusion:**

Studies using shorter observation periods will overestimate overdiagnosis by inclusion of cancers diagnosed early due to lead time among the nominally overdiagnosed tumours.

## Introduction

The issue of overdiagnosis in breast cancer screening is a topic of much interest and controversy [1-3]. Overdiagnosis is usually defined as the diagnosis as a result of screening of cancer that would never have been diagnosed in the woman's life in the absence of screening [1]. In theory, overdiagnosis can be estimated by comparison of incidence in a randomized trial of screening, but this would require that the control group was never screened and that both intervention and control groups were followed up to expiry or for more than at least two decades. In practice, none of the trials satisfy both of these criteria [4]. Consequently, overdiagnosis rates are usually estimated on the basis of changes in breast cancer incidence following the introduction of screening services in a population setting [1-3,5,6]. There is no uniform method of estimation, and, consequently, estimates vary considerably, from less than 5% to around 50% [1].

One of the first observable effects of breast screening is **the capability **to diagnose cancer before it would have occurred symptomatically, since screening works by diagnosing breast cancer at an earlier and more treatable stage [7]. The temporal advance in the time of diagnosis is known as the lead time. A major complication of estimating overdiagnosis is taking account of lead time to disentangle overdiagnosed from early-diagnosed cases [1]. Overdiagnosis can be thought of as cases whose lead time exceeds their remaining years of life. Cases whose lead time does not exceed their future lifetime will cause an increase in incidence to be observed but should not be included in the estimation of overdiagnosis. There is disagreement about the importance of lead time [8,9], but there is no doubt that the phenomenon exists, since incidence of breast cancer after a screen is considerably lower than in the absence of screening, indicating that many tumors have their period of diagnosis advanced [10].

In this article, we generate a set of breast cancer incidence figures, by individual year of age and individual calendar year, in the absence of screening. These are based approximately on the population of age 50 to 79 and the age-specific incidence of breast cancer in the late 1980s in the UK, just before the screening program started. We then generate the effect on occurrence of breast cancer of a 2-yearly screening program from age 50 to 69, assuming only lead time and no overdiagnosis. We use the difference between the two to evaluate the reliability of different strategies for adjusting for lead time in estimation of overdiagnosis.

## Materials and methods

We postulated a population of age 50 to 79 and of size and structure similar to those prevailing in the female population in England and Wales in the late 1980s [11,12]. We further postulated that invasive breast cancer incidence rates in the absence of screening would increase from 158.4 per 100,000 person-years at age 50 to 272.7 per 100,000 at age 79, again corresponding roughly to those prevailing in England and Wales in the years immediately prior to the screening program [11,12]. The population, incidence rates, and numbers of cases are shown by individual year of age in Table 1. For simplicity, we assumed that in the absence of screening, the population age structure and age-specific breast cancer incidence rates remain stable for the following 30 years. Thus, the same number of cases in each individual year of age occurred for the following 30 years.

**Table 1:** Postulated incidence of breast cancer by individual year of age in the absence of screening

Age, years	Population	Incidence, per 100,000	Number of cases
50	253,157	158.4	401

51	252,774	162.2	410

52	253,012	166.0	420

53	253,239	169.8	430

54	253,026	173.5	439

55	262,267	177.3	465

56	261,879	181.0	474

57	261,905	184.8	484

58	261,780	191.0	500

59	262,170	197.2	517

60	275,947	203.3	561

61	275,895	209.5	578

62	275,846	215.7	595

63	276,020	218.1	602

64	276,191	220.5	609

65	231,943	222.9	517

66	232,135	225.3	523

67	231,884	227.7	528

68	232,059	232.7	540

69	231,805	237.7	551

70	231,974	242.7	563

71	232,136	247.7	575

72	231,896	252.7	586

73	232,003	255.6	593

74	231,811	258.4	599

75	189,820	261.3	496

76	190,080	264.1	502

77	189,888	267.0	507

78	190,070	269.9	513

79	189,952	272.7	518

We then assumed the introduction of a screening program with 2-yearly screening applied to all women currently 50 to 69 years old, at years 1, 3, 5, and so on up to year 19 (that is, 10 rounds of screening in all). We assumed that the only effect of screening on incidence of breast cancer was one of lead time. We postulated that in screening a population age x in year y we would find 86% of the cancers which would have occurred in age x+1, year y+1, 64% of tumors which would have occurred in age x+2, year y+2, and so on up to 7% of the tumors from age x+10, y+11, as shown in Table 2. These figures correspond to an average lead time of around 40 months, which is consistent with the estimates for this age group [13-16], most of which also found screening sensitivities of close to 100%. For mathematical details, see the Appendix.

**Table 2:** Proportion of cancers with time of diagnosis advanced to the year of screening, by year*

Years after screen	Percentage of diagnoses advanced to screen year
1	86

2	64

3	48

4	35

5	26

6	20

7	15

8	11

9	9

10	7

By year at which diagnosis would have taken place in the absence of screening

We then compared the numbers of cancers between the screening and no-screening scenarios, for different age groups and periods of observation, to estimate the excess incidence that would be observed as a result of lead time alone and to evaluate the likely efficacy of various methods of taking account of lead time. We also calculated the resulting estimates for different strategies used in the past to take lead time into account. These include considering incidence up to 5 and 10 years after the upper age limit for screening and comparing breast cancer incidence in the screened population with the corresponding incidence of subjects in the unscreened population who are 2 to 5 years older [5,6]. This was solely a modeling study that involved no experimental work on human or animal subjects. No ethical approval or consent was required.

## Results

Table 3 shows the numbers of cancers diagnosed over 30 years, by calendar year and year of age, under the scenario of 2-yearly screening of women 50 to 69 years old for 10 rounds (the first 20 years) as described above. The corresponding numbers of cancers in the absence of screening would simply be the column of cancers in Table 1, 401, 410, and so on, repeated 30 times. The lead-time effect can be seen for age 50, year 1, for example, as

**Table 3:** Numbers of tumors diagnosed by age and year in a 2-yearly screening program

Age, years	Calendar year																													
	**1**	**2**	**3**	**4**	**5**	**6**	**7**	**8**	**9**	**10**	**11**	**12**	**13**	**14**	**15**	**16**	**17**	**18**	**19**	**20**	**21**	**22**	**23**	**24**	**25**	**26**	**27**	**28**	**29**	**30**

50	1,812	401	1,812	401	1,812	401	1,812	401	1,812	401	1,812	401	1,812	401	1,812	401	1,812	401	1,812	401	401	401	401	401	401	401	401	401	401	401

51	1,863	57	1,863	57	1,863	57	1,863	57	1,863	57	1,863	57	1,863	57	1,863	57	1,863	57	1,863	57	410	410	410	410	410	410	410	410	410	410

52	1,919	59	1,242	59	1,242	59	1,242	59	1,242	59	1,242	59	1,242	59	1,242	59	1,242	59	1,242	59	151	420	420	420	420	420	420	420	420	420

53	1,979	60	1,282	31	1,282	31	1,282	31	1,282	31	1,282	31	1,282	31	1,282	31	1,282	31	1,282	31	155	224	430	430	430	430	430	430	430	430

54	2,045	61	1,326	32	1,119	32	1,119	32	1,119	32	1,119	32	1,119	32	1,119	32	1,119	32	1,119	32	103	228	285	439	439	439	439	439	439	439

55	2,111	65	1,364	34	1,149	25	1,149	25	1,149	25	1,149	25	1,149	25	1,149	25	1,149	25	1,149	25	109	179	302	344	465	465	465	465	465	465

56	2,164	66	1,397	35	1,176	26	1,087	26	1,087	26	1,087	26	1,087	26	1,087	26	1,087	26	1,087	26	89	182	246	351	379	474	474	474	474	474

57	2,224	68	1,433	35	1,204	26	1,112	22	1,112	22	1,112	22	1,112	22	1,112	22	1,112	22	1,112	22	91	158	252	304	387	411	484	484	484	484

58	2,288	70	1,467	36	1,229	27	1,133	23	1,098	23	1,098	23	1,098	23	1,098	23	1,098	23	1,098	23	83	164	231	315	356	425	445	500	500	500

59	2,351	72	1,497	38	1,249	28	1,149	24	1,113	22	1,113	22	1,113	22	1,113	22	1,113	22	1,113	22	86	154	239	296	368	400	460	470	517	517

60	2,408	79	1,516	41	1,261	30	1,157	26	1,119	23	1,112	23	1,112	23	1,112	23	1,112	23	1,112	23	87	167	241	321	371	434	464	511	522	561

61	2,422	81	1,516	42	1,259	31	1,155	26	1,115	24	1,108	24	1,108	24	1,108	24	1,108	24	1,108	24	90	172	249	331	383	447	478	526	538	578

62	2,416	83	1,505	43	1,249	32	1,146	27	1,105	25	1,098	25	1,098	25	1,098	25	1,098	25	1,098	25	92	177	256	341	394	460	492	541	553	595

63	2,385	84	1,485	44	1,234	32	1,133	28	1,094	25	1,087	25	1,087	25	1,087	25	1,087	25	1,087	25	93	179	259	345	399	466	498	548	560	602

64	2,333	85	1,462	44	1,219	33	1,122	28	1,086	25	1,079	25	1,079	25	1,079	25	1,079	25	1,079	25	94	181	262	349	403	471	504	554	566	609

65	2,260	72	1,442	38	1,208	28	1,114	24	1,079	22	1,073	22	1,073	22	1,073	22	1,073	22	1,073	22	80	154	223	296	342	400	428	470	481	517

66	2,286	73	1,456	38	1,218	28	1,123	24	1,088	22	1,081	22	1,081	22	1,081	22	1,081	22	1,081	22	81	156	225	299	346	405	433	476	486	523

67	2,313	74	1,470	38	1,228	28	1,131	24	1,095	22	1,089	22	1,089	22	1,089	22	1,089	22	1,089	22	82	157	227	302	350	408	437	480	491	528

68	2,340	76	1,482	39	1,235	29	1,136	25	1,099	23	1,093	23	1,093	23	1,093	23	1,093	23	1,093	23	84	161	232	309	358	418	447	491	502	540

69	2,360	77	1,487	40	1,237	30	1,136	25	1,098	23	1,092	23	1,092	23	1,092	23	1,092	23	1,092	23	85	164	237	315	365	426	456	501	512	551

70	563	79	203	41	132	30	105	26	94	23	87	23	87	23	87	23	87	23	87	23	87	168	242	322	373	435	466	512	524	563

71	575	575	207	299	135	221	108	188	96	171	89	171	247	329	381	445	89	171	89	171	89	171	247	329	381	445	476	523	535	575

72	586	586	586	305	381	225	305	192	271	174	252	174	252	335	388	453	485	174	252	174	252	174	252	335	388	453	485	533	545	586

73	593	593	593	593	385	439	308	373	274	339	255	339	255	339	393	459	491	540	255	339	255	339	255	339	393	459	491	540	551	593

74	599	599	599	599	599	443	479	377	426	343	397	343	397	343	397	463	496	545	557	343	397	343	397	343	397	463	496	545	557	599

75	496	496	496	496	496	496	397	422	353	384	328	384	328	384	328	384	411	451	461	496	328	384	328	384	328	384	411	451	461	496

76	502	502	502	502	502	502	502	427	447	388	416	388	416	388	416	388	416	457	467	502	502	388	416	388	416	388	416	457	467	502

77	507	507	507	507	507	507	507	507	451	461	420	461	420	461	420	461	420	461	472	507	507	507	420	461	420	461	420	461	472	507

78	513	513	513	513	513	513	513	513	513	467	477	467	477	467	477	467	477	467	477	513	513	513	513	467	477	467	477	467	477	513

79	518	518	518	518	518	518	518	518	518	518	482	518	482	518	482	518	482	518	482	518	518	518	518	518	482	518	482	518	482	518

410+0.86×410+0.64×420+⋯+0.07×561=1,812.

**Figure 1:**
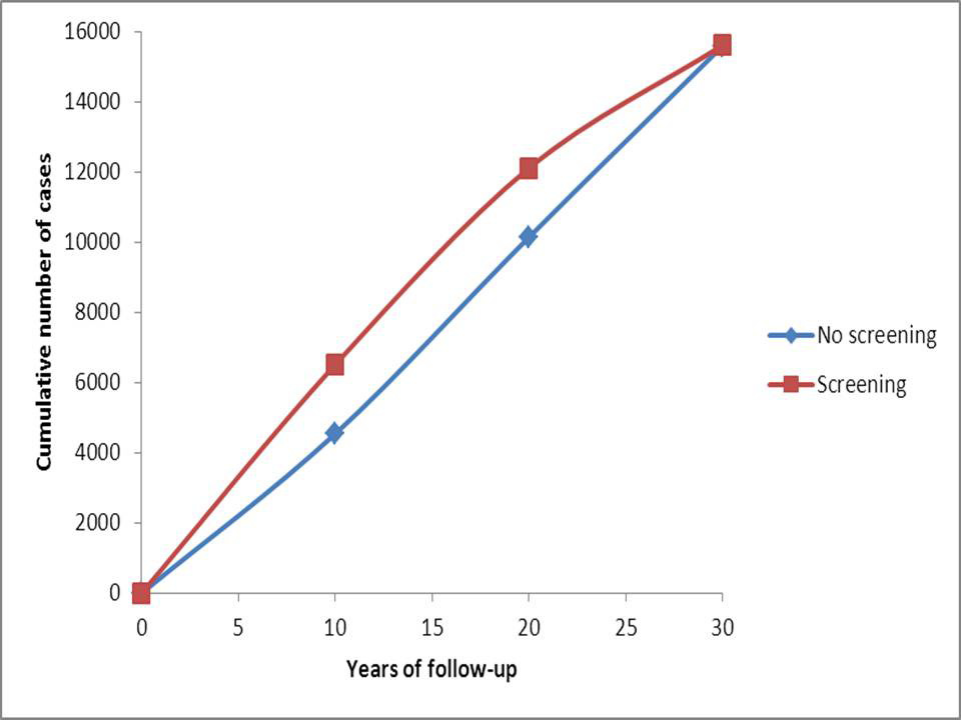
**Expected cumulative incidence over ages 50 to 79 in a cohort of women of age 50 at the start, with and without 2-yearly screening from age 50 to 69**.

Thus, the effect of lead time is to shift cancers along the diagonal of the table, upward and leftward. This reflects the fact that earlier detection brings forward the diagnosis of the tumor both in calendar period and age. For example, without screening in year 1, there would be 4,540 cancers diagnosed at ages 50 to 59; with screening, there would be 20,756. Conversely, in year 10 at ages 70 to 79, there would be 5,452 cancers diagnosed without screening but only 3,268 with screening. This shift in age and time of diagnosis also means that if incidence is increasing with age, with time, or with both, then the observed excess due to lead time will be larger. Figure 1 shows the cumulative numbers of breast cancer cases observed in the cohort of age 50 at year 1, over 30 years, with and without screening. The incidences separate dramatically, in the first 10 years, and the disparity remains roughly constant over the following 10 years; then the two cumulative graphs come together at 30 years. This demonstrates that the only phenomenon generated by the screening in this scenario is lead time. The screening diagnoses many cancers earlier than they would have been diagnosed in the absence of screening, but after the screening stops, there is a deficit of cases in the screened group and the unscreened group 'catches up'.

Some researchers attempt to control for lead time by comparing incidence up to 10 years above the upper age limit for screening [5]. The rationale for this **approach **is that an excess of cases observed at screening ages because of lead time will be balanced by a corresponding deficit in cases above the screening age range. Table 4 shows the cumulative numbers of cancers with and without screening, by years of follow-up and upper age limit. Clearly, when the age range for screening alone is observed, lead time confers a substantial increase in observed cases, which remains substantial 10 years after the program has stopped. From lead time alone, 20-year observation at ages 50 to 69 would confer an observed excess incidence of 37% in the screened population. The excess would only fall below 10% with a follow-up of 25 years or more. For the excess to be nullified, or almost nullified, we would require a 30-year follow-up, including observation up to 10 years above the upper age limit for screening. This is because lead time shifts the diagnosis not only in terms of age of the subject but also in terms of the calendar year of diagnosis.

**Table 4:** Cumulative numbers of breast cancer cases observed with and without screening

Age range observed, years	Years of follow-up	Number of cases		Observed percentage excess
		
		Without screening	With screening	incidence With screening
50-69	5	50,270	102,380	102
	
	10	101,440	153,460	51
	
	15	152,160	226,680	49
	
	20	202,880	277,060	37
	
	25	253,600	304,080	20
	
	30	304,320	351,260	15

50-74	5	65,300	113,400	74
	
	10	130,600	170,500	31
	
	15	195,900	250,100	28
	
	20	261,200	307,675	18
	
	25	326,500	341,975	5
	
	30	391,800	402,100	3

50-79	5	77,980	126,069	62
	
	10	155,960	195,018	25
	
	15	233,940	285,435	22
	
	20	311,920	354,714	14
	
	25	389,900	400,239	3
	
	30	467,880	472,014	1

Another strategy sometimes employed to account for lead time is to compare the incidence in the screened population with the corresponding incidence of subjects in the unscreened population who are 2 to 5 years older [5,6]. Table 5 shows the incidence per 100,000 person-years in 5-year age groups and time periods in the screened and unscreened populations. Note that because alternate 5-year periods contain two screens and three screens, the overall incidence in the screened population fluctuates from period to period. Note also that within 5-year periods there is no monotonic increase in incidence with age, because some of the older age groups have cases shifted to younger groups because of lead time. If we compare the cumulative incidence over 10 years between the age group of 50 to 69 in the screened population and the age group of 55 to 74 in the unscreened population, using Table 5 data, we would observe 3,000 cases per 100,000 women in the screened population and 2,190 in the latter, an excess of 37%. At 20 years, we would observe 5,415 cases per 100,000 women in the screened population and 4,380 in the unscreened population, an excess of 24%. At 30 years, the cumulative incidence figures would be 6,865 and 6,570, an excess of 4%.

**Table 5:** Incidence rates per 100,000 person-years in the screened and unscreened populations

Age	Population	Calendar period, years					
		
group, years		1-5	6-10	11-15	16-20	21-25	26-30
50-54	Screened	406	259	365	259	143	166
	
	Unscreened	166	166	166	166	166	166

55-59	Screened	379	176	258	175	94	180
	
	Unscreened	186	186	186	186	186	186

60-64	Screened	381	169	242	164	91	190
	
	Unscreened	213	213	213	213	213	213

65-69	Screened	441	196	285	193	97	204
	
	Unscreened	229	229	229	229	229	229

70-74	Screened	190	104	110	125	125	223
	
	Unscreened	251	251	251	251	251	251

75-79	Screened	267	250	228	246	236	246
	
	Unscreened	267	267	267	267	267	267

**Figure 2:**
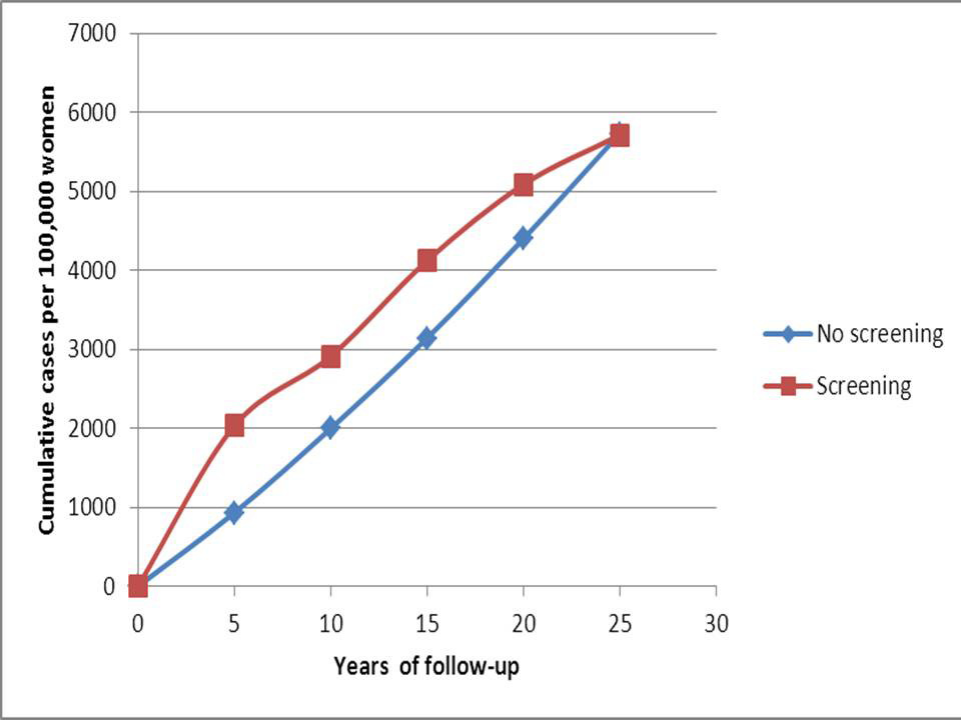
**Expected cumulative incidence over 25 years in a screened group of ages 50 to 54 and in an unscreened group of age 55 to 59 at recruitment at the start of follow-up**.

A more exact 'aging' adjustment for lead time might be to compare the cumulative incidence of a screened cohort with that of an unscreened cohort that is 5 years older. Figure 2 shows the cumulative incidence per 100,000 women up to year 25 (not 30, since we do not have data for ages 80 and above) for ages 50 to 54 at the start of the follow-up period in the screened population and 55 to 59 in the unscreened population. The incidence curves come together only at 25 years. There is still a substantial excess in the screened population at 20 years.

## Discussion

We posited a population of age 50 to 79 with age distribution and breast cancer incidence similar to those that prevailed in the UK before the NHS Breast Screening Programme was initiated. We assumed that age-specific incidence in an unscreened population would remain unchanged over 30 years. We then imposed a screening program with 2-yearly screening for 20 years from age 50 to 69 (as is common in Europe). We calculated the incidence in the screened population, assuming that the only influence of screening on incidence was lead time, using lead-time parameters similar to those observed in a large breast screening trial [13,14]. Thus, we calculated the expected effects on incidence from lead time alone, with no influence of overdiagnosis or changes in underlying risk.

The most important finding was that lead time can be expected to add substantially to observed incidence in a screened population. Although the increase is greatest in the early years of the program, there is still a substantial excess at long-term follow-up. From Table 4, in the screened age group, 50 to 69, a 37% excess cumulative incidence was apparent at 20 years, and a 15% excess at 30 years, 10 years after the program stopped. Thus, the first conclusions from this work must be that lead time contributes a considerable observed increase in incidence in screening age groups and that for an estimate of overdiagnosis to be reliable, it must correct for lead time.

Secondly, the results give us some qualifications to the use of common methods of correction for lead time and suggest that correction for lead time in our estimates and those of others may be inadequate [2,3,5,6]. The method of considering incidence up to an age in excess of the upper age limit for screening can be reliable but only if more than two decades of follow-up are available. From Table 4, with incidence to age 74, the excess from lead time alone at 20 years was 18%. With incidence to age 79, the excess at 20 years was 14%. It is only at 25 to 30 years of follow-up that the excess incidence decreased to a figure close to zero.

It should be noted that our estimates pertain to a population that is 100% screened. In the screening trials, results are usually given for the group randomly assigned to the offer of screening, which will contain some non-attenders. Both lead-time effects and overdiagnosis will be smaller in an invited group, and the attenuation depends on the proportion of non-attenders.

At the end of 10 years, from lead time alone there was an excess cumulative incidence at ages 50 to 79 of 25% in the screened population. Kalager and colleagues [5], who used this method of controlling for lead time, observed an 18% excess incidence in the Norwegian breast screening program at approximately 10 years. With 77% participation in the Norwegian program [5], one would expect 19% (0.77 × 25%) at 10 years. Thus, all of the excess incidence in that study might be explained by lead time. The excess incidence in the screened ages at a follow-up of only 10 years amounts to 52,020 cases, whereas the deficit above the screened ages is only 12,962 cases (Table 4). In the age group of 70 to 79 in the first 10 years, 70% of the cancers diagnosed are in women who have never been screened (Table 3). Our study [3] had a 15-year follow-up, but even at 15 years, our results here indicate that there would still be residual lead-time effects when this type of correction is used.

We assumed that the incidence rates would have remained stable over time in the absence of screening, for simplicity of calculation. If we had assumed that underlying incidence rates were increasing, as indeed they were almost everywhere in the world in the late 20th century, the effect of lead time and the potential to overestimate overdiagnosis would have been even greater.

Our lead-time model is based on the exponential sojourn time model of Walter and Day [15], whose article gives a full discussion of the mathematical details and modeling assumptions involved in estimating cancer screening parameters. Walter and Day gave a range of estimates of the mean preclinical screen detectable period from 1.7 to 3.1 years; however, more recent estimates have been close to our estimate of 40 months [13-16].

The recent UK review of breast cancer screening estimated overdiagnosis of 10% to 20% depending on the denominator used, from the difference between study and control group incidence in three of the randomized screening trials [16]. For two of the trials, the incidence extended only 6 years beyond the end of screening in the study group, so the overdiagnosis estimates in the review are also likely to be overestimates.

Similarly, the attempt by Kalager and colleagues [5] to control for lead time by comparing the screened population with subjects in the unscreened population who are 2 to 5 years older is inadequate for adjusting for lead time. Our results show that at 10 years, even with the 5-year adjustment, lead time would confer a 37% increase in incidence. Correcting for the participation rate of 77%, we would expect a 28% increase in incidence, and the increase observed by Kalager and colleagues [5] was actually smaller than this (18%).

Morrell and colleagues [6] applied a similar 5-year aging correction for lead time in the New South Wales breast screening program and observed a 30% excess incidence after adjustment, and at about 10 years after the program started, with 60% participation. From lead time alone, we would expect a 22% increase (0.6 × 37%), suggesting that overdiagnosis accounts for an 8% excess and that the remainder of the 30% is a residual lead-time effect. It is also worth noting that the estimate by Morell and colleagues [6] is not based on cumulative incidence but only on the three years from 1999 to 2001 when the program was mature. More importantly, it is worth noting that the estimate of more than 30% overdiagnosis in the UK program by Jørgensen and Gøtzsche [2] has no adjustment for lead time at all and therefore cannot be regarded as a reliable estimate of overdiagnosis.

Further empirical evidence that overdiagnosis is a smaller problem than generally thought comes from the Swedish Two-County Trial of breast cancer screening. In one county, the cumulative incidence over 29 years was identical between study and control groups [17]. Since the control group was subject to screening 7 years later than the study group, this does not rule out overdiagnosis, but the fact that a population experiencing an additional 100,000 screening episodes had no increase whatsoever in incidence suggests that overdiagnosis is a minor phenomenon.

It should be noted that our estimates are based on incidence of invasive cancer only. We have blocked the time into discrete years and posited simultaneous screening of all subjects rather than a screening round taking several months to cover the population. Also, it could be argued that the average sojourn time (the duration of the preclinical screen detectable period), and therefore lead time, is smaller than used in our estimates. We did, however, use an estimate of just over 3 years, which is commonly observed for ages of 50 or more [13,14,18,19]. Indeed, some colleagues have estimated considerably greater sojourn times, albeit with lesser sensitivity [20]. Also, the general point will still hold that lead time confers an excess incidence even with the common corrections used up to 20 years after the program starts. Improvements to screening technology may change the sojourn time and sensitivity parameters. However, they are likely to increase rather than reduce lead time, so the qualitative implications of our results will remain relevant. It should also be noted that although we posited an average lead time of 40 months, the range of individual values is wide. For example, 35% of cancers that would have occurred 3 to 4 years after a screen have their diagnosis advanced to the screening year.

We estimated effects on incidence of lead time alone. Of course, there will be other influences, including changes in underlying incidence because of trends in risk factor exposure and because of overdiagnosis. However, our results show that estimation of either of the latter will be contaminated by lead time unless there is very long (greater than two decades) observation. The major implication of this is that it is easy to overestimate overdiagnosis, particularly in uncontrolled observation of incidence before and after screening. To avoid such overestimation, it is necessary to estimate lead time explicitly or to have a very long period of observation. To conclude that large numbers of breast cancers would never progress in the absence of treatment is a major biological leap of faith, and every effort should be made to rule out alternative explanations of increases in breast cancer incidence.

## Conclusions

The results here indicate that to estimate overdiagnosis using either the compensatory drop above the age range for screening or an age inflation, very-long-term follow-up is required to avoid bias from residual lead-time effects. Previous estimates of overdiagnosis are likely to be overestimates.

## Appendix

### Estimation of proportions of cancers with diagnosis time shifted by screening, by year since screen

Let λ_0 _be the instantaneous rate of transition from no detectable disease to presymptomatic screen-detectable breast cancer, and λ_1 _the subsequent rate of transition to symptomatic disease. With an exponential distribution of time to each transition [13], the probability of clinical symptomatic disease between time 0 and time 1 year, in a person screened negative at time 0, is

$$P_{1}=\overset{1}{\underset{0}{\int }}\lambda_{0}e^{-\lambda_{0}u}\left(1-e^{-\lambda_{1}\left(1-u\right)}\right)du=1-e^{-\lambda_{0}}-\lambda_{0}e^{-\lambda_{1}}\overset{1}{\underset{0}{\int }}e^{\left(\lambda_{1}-\lambda_{0}\right)u}du$$

$$=1-e^{-\lambda_{0}}-\frac{\lambda_{0}\left(e^{-\lambda_{0}}-e^{-\lambda_{1}}\right)}{\left(\lambda_{1}-\lambda_{0}\right)}$$

This is essentially the probability of transition from no detectable disease to presymptomatic including the possibilities of remaining presymptomatic or progressing to symptomatic disease MINUS the probability of transition only to the presymptomatic phase. This difference is equal to the probability of transition from no detectable disease to symptomatic disease. Suppose an incidence of 2 per thousand per year. The corresponding value for λ_0 _would be 0.002 (corresponding to an incidence of 2 per thousand person-years) and λ_1 _= 0.3 (corresponding to a mean sojourn time of 40 months), this probability is 0.000272, 14% of the expected incidence (0.000272/0.002 = 0.14). Thus 86% of the tumours expected to arise in the first year after screening have their diagnosis advanced to the time of the screen.

The probability of symptomatic incidence in years 0 to 2 is correspondingly

$$P_{2}=1-e^{-2\lambda_{0}}-\frac{\lambda_{0}\left(e^{-2\lambda_{0}}-e^{-2\lambda_{1}}\right)}{\left(\lambda_{1}-\lambda_{0}\right)}$$

With λ_0 _= 0.002 and λ_1 _= 0.3, this would be 0.000991. Therefore, the probability of symptomatic occurrence between 1 and 2 years after the screen would be 0.000991-0.000272 = 0.000719, 36% of the expected incidence in the absence of screening. Thus 64% of the tumours which would have arisen in the second year after screening have their diagnosis advanced to the time of the screen. Proportions for subsequent years are calculated similarly.

## Competing interests

The authors declare that they have no competing interests.

## Authors' contributions

SWD was responsible for the concept of the study and for the mathematical formulation and contributed to drafting the manuscript. DP was responsible for informatics and computing modeling and contributed to drafting the manuscript. Both authors read and approved the final manuscript.
